# Antioxidant Therapy and Antioxidant-Related Bionanomaterials in Diabetic Wound Healing

**DOI:** 10.3389/fbioe.2021.707479

**Published:** 2021-06-24

**Authors:** Wenqian Zhang, Lang Chen, Yuan Xiong, Adriana C. Panayi, Abudula Abududilibaier, Yiqiang Hu, Chenyan Yu, Wu Zhou, Yun Sun, Mengfei Liu, Hang Xue, Liangcong Hu, Chenchen Yan, Xuedong Xie, Ze Lin, Faqi Cao, Bobin Mi, Guohui Liu

**Affiliations:** ^1^Department of Orthopaedics, Union Hospital, Tongji Medical College, Huazhong University of Science and Technology, Wuhan, China; ^2^Hubei Province Key Laboratory of Oral and Maxillofacial Development and Regeneration, Wuhan, China; ^3^Division of Plastic Surgery, Brigham and Women’s Hospital and Harvard Medical School, Boston, MA, United States; ^4^Department of Neurosurgery, Union Hospital, Tongji Medical College, Huazhong University of Science and Technology, Wuhan, China

**Keywords:** wound healing, antioxidative therapy, oxidative stress, bionanomaterials, diabetes mellitus

## Abstract

Ulcers are a lower-extremity complication of diabetes with high recurrence rates. Oxidative stress has been identified as a key factor in impaired diabetic wound healing. Hyperglycemia induces an accumulation of intracellular reactive oxygen species (ROS) and advanced glycation end products, activation of intracellular metabolic pathways, such as the polyol pathway, and PKC signaling leading to suppression of antioxidant enzymes and compounds. Excessive and uncontrolled oxidative stress impairs the function of cells involved in the wound healing process, resulting in chronic non-healing wounds. Given the central role of oxidative stress in the pathology of diabetic ulcers, we performed a comprehensive review on the mechanism of oxidative stress in diabetic wound healing, focusing on the progress of antioxidant therapeutics. We summarize the antioxidant therapies proposed in the past 5 years for use in diabetic wound healing, including Nrf2- and NFκB-pathway-related antioxidant therapy, vitamins, enzymes, hormones, medicinal plants, and biological materials.

## Introduction

Type II Diabetes (T2DM) is characterized by chronic hyperglycemia and is associated with significant vasculopathy (Brownlee, 2001). T2DM is a worldwide healthcare problem with increasing global prevalence (NCD Risk Factor Collaboration (Ncd-RisC), 2016). Foot ulcers, a lower-extremity complication of T2DM with high recurrence rates, is a substantial burden for patients with T2DM and society as a whole (Armstrong et al., 2017). The lifetime risk of a patient with T2DM developing a foot ulcer is estimated to be as high as 25% (Singh et al., 2005), and it is believed that every 30 s worldwide a lower limb is amputated as a consequence of T2DM (Boulton et al., 2005). An effective therapeutic approach that can improve wound healing in T2DM has the potential to revolutionize medicine.

Wound healing is the precise interplay of complex biological and molecular events including cell migration, cell proliferation, and extracellular matrix (ECM) deposition. The process is classically divided into four overlapping phases of coagulation, inflammation, migration-proliferation (including matrix deposition), and remodeling (Falanga, 2005). Wound healing requires the combined function of different cells including platelets, neutrophils, monocytes, macrophages, endothelial cells, keratinocytes, fibroblasts, and myofibroblasts. However, these processes and healing stages have been shown to be dysfunctional in patients with T2DM and collectively lead to overall impaired healing of acute wounds predisposing the patients to chronic, non-healing wounds such as diabetic foot ulcers (Avishai et al., 2017).

Oxidative stress is one of the main mechanisms involved in chronic diabetic foot ulcers. The concept of oxidative stress was introduced by H. Sies in 1985, who defined it as a shift in the prooxidant-antioxidant balance in favor of the former. Oxidative stress now also refers to an imbalance between oxidants and antioxidants in favor of oxidants, leading to a disruption of redox signaling and molecular damage (Sies, 2015). Oxidative stress can be classified into different subgroups ranging from physiological oxidative stress to excessive and toxic oxidative burden (Lushchak, 2014). Oxygen-dependent redox-sensitive signaling processes are an integral component of the healing cascade. Oxidative stress is necessary for wound disinfection and promotes wound healing by facilitating hemostasis, inflammation, angiogenesis, granulation tissue formation, wound closure, and development and maturation of the extracellular matrix(ECM) (Schafer and Werner, 2008; Sen and Roy, 2008). However, excessive and uncontrolled oxidative stress results in sustaining and deregulating inflammation, playing a central role in the pathogenesis of chronic non-healing wounds (Bryan et al., 2012). In diabetic wounds, ROS production through several ROS-generating enzymes is elevated, resulting in impaired wound healing processes via increased cell apoptosis and senescence with ongoing oxidative stress, lipid peroxidation, protein modification, and DNA damage (Schafer and Werner, 2008).

Given the importance of oxidative stress in the pathology of diabetic ulcers, this comprehensive review will focus on oxidative stress in diabetic wound healing, paying particular attention to the progress of antioxidant therapies in the past 5 years (Table 1).

**TABLE 1:** Antioxidant therapies used in diabetic wounds reported in the past 5 years (2016–2020).

**Category**	**Therapy**	**Mechanism**	**Effects**	**References**
Nrf2-pathway-related	siKeap1	Inhibit Keap1, increase Nrf2 nuclear translocation	Accelerate wound healing, improve redox homeostasis, and promote angiogenesis	Soares et al., 2016
	Lipoproteoplex	Deliver siKeap1	Restore Nrf2 antioxidant function, Accelerate wound healing, augment reduction-oxidation homeostasis	Rabbani et al., 2017
	Exosomes from ADSCs overexpressing Nrf2	Increase Nrf2 level	Prevent senescence of EPCs, inhibit ROS and inflammatory cytokine formation and promote angiogenesis	Li et al., 2018b
NFκB-pathway-related	miR-146a	Decrease levels of phosphorylated IκB-α, phosphorylated NFκB, and total NFκB	/	Zgheib et al., 2019; Niemiec et al., 2020; Sener et al., 2020
	SRT1720	Activate SIRT1	Accelerate wound healing and promote angiogenesis	Li et al., 2019;
Vitamins	Vitamins E and C	Restore the antioxidant enzyme activities, reduce ROS levels	Accelerate wound healing	Pessoa et al., 2016
	Mono-epoxy-tocotrienol-α	Antioxidant effects	Increase the expression of genes involved in cell growth, motility, angiogenesis and mitochondrial function	Xu et al., 2017
	Folic acid	Suppress oxidative stress	Accelerate wound healing and promote collagen deposition	Zhao et al., 2018
	SkQ1	Suppress mitochondrial ROS production	Accelerate wound healing and promote epithelization, granulation tissue formation, and angiogenesis	Demyanenko et al., 2017
Enzymes	SOD	Catalyze the decomposition of superoxide radicals into hydrogen peroxide	Accelerate wound healing	Zhang et al., 2018
	HO-1	Cleaves the α-methene bridges of heme to produce equimolar amounts of biliverdin and carbon monoxide	Inhibit inflammatory cytokine formation, increase antioxidants and promote angiogenesis	Chen et al., 2016b; Kumar et al., 2019
Hormones	17β-estradiol	Regulate energy homeostasis and glucose metabolism	Reduce excessive ROS formation and facilitate cell survival	Oh et al., 2019
	5α-dihydrotestosterone	Regulate energy homeostasis and glucose metabolism	Increase the proportion of type I and type III collagen fibers and superoxide dismutase levels	Goncalves et al., 2016
Medicinal plants	Dimethyl fumarate	Activate Nrf2	Decrease oxidative damage and inflammation, and accelerate wound healing	Li et al., 2018b
	RTA 408	Upregulate expression of Nrf2 target genes	Accelerate wound healing and promote re-epithelialization	Rabbani et al., 2018
	Genistein	Activate Nrf2, downregulate NFκB	Accelerate wound healing	Eo et al., 2016
	Asiatic acid	Downregulate NFκB activation and reduce pro-inflammatory cytokines	Attenuate prolonged inflammation and Accelerate wound healing	Han et al., 2019
	Syringic acid	Suppress NFκB activation and the inflammatory response	Accelerate wound healing	Ren et al., 2019
	Hydroethanolic extract of strychnos pseudoquina	Modulate oxidative status and microstructural reorganization	Accelerate wound healing	Sarandy et al., 2017
	Deoxyshikonin	Exert antioxidant activity and promote phosphorylation of ERK and p38 and VEGFR-2 expression	Accelerate wound healing	Park et al., 2018
	Quercetin	Suppress oxidative stress and enhance the antioxidant defense system	Accelerate wound healing	Ahmed et al., 2018
Biological materials	SOD-loaded hydrogel	Sustained release of SOD with high activity	Promote re-epithelialization and collagen deposition	Zhang et al., 2018
	CONP-loaded GelMA hydrogel	Uptake exudate, scavenge free radicals	Accelerate wound healing	Augustine et al., 2021
	Edaravone-loaded alginate-based nanocomposite hydrogel	Downregulate ROS levels	Accelerate wound healing	Fan et al., 2019
	Injectable, self-healable zwitterionic cryogel	Sustained release of miRNA-146a-CNPs	Accelerate wound healing	Sener et al., 2020
	Nanosilk	Deliver CNP-miR146a to the wound bed	Downregulate proinflammatory signaling and promote pro-fibrotic processes	Niemiec et al., 2020
	AA-PL scaffolds	Relieve the high oxidative stress, inflammation and infection	Promote angiogenesis, extracellular matrix formation and re-epithelization	Han et al., 2019
	tFNAs	Activate the Akt/Nrf2/HO-1 signaling pathway	Control inflammation, prevent oxidative damage, facilitate angiogenesis	Lin et al., 2020
	Berberine nanohydrogel	Activate SIRT1, inhibit the expression of NFκB	Accelerate wound healing, reduce inflammation, promote angiogenesis	Zhang et al., 2020

## Oxidative Stress in Wound Healing

### Normal Wound Healing

Wound healing is a precise integration of complex biological and molecular events including cell migration, cell proliferation, and ECM deposition. It can be divided into the four overlapping phases of coagulation, inflammation, migration-proliferation (including matrix deposition), and remodeling (Falanga, 2005). As has been reported in numerous studies, wound healing is the joint effort of various cells including platelets, neutrophils, monocytes, macrophages, endothelial cells, keratinocytes, fibroblasts, and myofibroblasts.

In the coagulation phase, platelets, together with a meshwork of polymerized fibrinogen (fibrin), fibronectin, vitronectin, and thrombospondin, participate in the construction of fibrin plugs, providing a temporary wound coverage to protect the wound and defend bacteria (Singer and Clark, 1999). During their incorporation within the plug, platelets aggregate and release a wide range of growth factors such as platelet-derived growth factor (PDGF), epidermal growth factor (EGF) and transforming growth factor-β (TGF-β) (Rodrigues et al., 2019).

During the inflammation phase, neutrophils and monocytes, recruited by PDGF, aid in microorganism killing and produce several key growth factors and mediators to promote wound healing (Singer and Clark, 1999). Neutrophils secrete proteolytic enzymes and release reactive oxygen species (ROS) into the wound bed to combat invading bacteria. Neutrophils also release important cytokines, such as TGF- β1, monocyte chemoattractant protein 1, and fragments of ECM proteins to recruit monocytes (Mirza and Koh, 2015). Monocytes, recruited to the wound, differentiate into pro-inflammatory macrophages to aid in the inflammatory process by phagocytosing dead neutrophils, cellular debris and bacteria (Schafer and Werner, 2008). These leukocytes also secrete new growth factors and cytokines such as TGF- β1, fibroblast growth factor (FGF), PDGF, and vascular endothelial growth factor (VEGF) aiding in the migration-proliferation phase (Mirza and Koh, 2015).

In the migration-proliferation phase, keratinocytes start migrating with the disassembly of hemidesmosomes, and a keratinocyte proliferative burst is followed to close the defect and create a new epidermis (re-epithelialization) (Martin, 1997). Fibroblasts convert into myofibroblasts that secrete ECM proteins, aiding in closure of the wound (Bainbridge, 2013; Quan et al., 2013). Endothelial cells also play a crucial role in this phase. Endothelial cells proliferate, migrate, and branch to form new blood vessels (Falanga, 2005), allowing re-supply of oxygen and other nutrients. While new blood vessels form, endothelial cells, together with macrophages and fibroblasts, form the early granulation tissue that begins the process of contraction (Martin, 1997). Due to expression of α- smooth muscle actin in microfilament bundles or stress fibers, myofibroblasts exhibit contractile properties, promoting contraction and maturation of the granulation tissue (Bainbridge, 2013). Granulation tissue lays down collagen, eventually resulting in scar formation (Bainbridge, 2013; Landen et al., 2016; Tracy et al., 2016).

The remodeling phase is the last phase in healing. In this phase, synthesis of ECM is considerably reduced, and synthesized components are modified as the matrix is remodeled. Excess cells undergo apoptosis and are removed by resident macrophages and histiocytes (Schafer and Werner, 2008). With the development of the recovery process, granulation tissue rich in type III collagen is replaced by a less vascularized and more resistant tissue rich in type I collagen (Singer and Clark, 1999). The newly formed tissue is strong due to the tensile strength of various components of the ECM and fibroblasts of the scar tissues (Stadelmann et al., 1998). Elastin, which is absent in the granulation tissue and normally contributes to skin elasticity, reappears in this phase (Bainbridge, 2013; Landen et al., 2016; Tracy et al., 2016). The skin barrier is re-established to protect the newly formed tissue from the environment (Loots et al., 1998). The remodeling phase proclaims the termination of wound healing.

### Oxidative Stress Impairs Diabetic Wound Healing

Individuals with diabetes demonstrate disturbances in all four individual healing stages that collectively lead to an overall impaired wound healing predisposing to chronic non-healing wounds such as diabetic foot ulcers (Avishai et al., 2017). Several studies have highlighted that impaired wound healing in diabetes is associated with the elevated levels of oxidative stress (Martin, 1996; Schafer and Werner, 2008). Hyperglycemia induces an increase in intracellular ROS generation, activating intracellular metabolic pathways, such as the polyol pathway and PKC signaling, leading to suppression of antioxidant enzymes and compounds (Evans et al., 2002). Excessive ROS generation in diabetes is also due to acute rises in serum glucose and accumulation of advanced glycation end products (AGEs) (Monnier et al., 2006). AGEs are potent prooxidants, a risk factor for injury and chronic ulcers. In chronic diabetic wounds, redox homeostasis is damaged by excessive ROS generation, causing loss of antioxidants and resulting in cell oxidative damage and wound healing inhibition (Wlaschek and Scharffetter-Kochanek, 2005; Zhang et al., 2018). The impacts of oxidative stress on the individual phase of wound healing are summarized as follows.

In the inflammation phase, oxidative stress in wounds influences the normal function of macrophages and neutrophils, resulting in prolonged inflammation. The increased number of neutrophils results in the production of ROS and excessive oxidative stress during inflammation, which damages the surrounding cells, tissues, and fibroblasts (Park et al., 2018). Oxidative stress also influences the macrophage differentiation and polarization impairing wound healing. T2DM induces pathological hematopoietic stem cells (HSCs) oxidant stress that can reduce the number and function of terminally differentiated inflammatory cells. Yan et al. (2018) reported that T2DM induces oxidant stress in HSC through a Nox-2-dependent mechanism and decreases microRNA let-7d-3p, which, in turn, upregulates the expression of Dnmt1. Increased Dnmt1 expression results in the downregulation of the genes responsible for HSC differentiation to monocytes/macrophages, and consequently reduces macrophage infiltration, driving polarization toward M1 macrophages (pro-inflammatory function), which causes excessive and prolonged inflammation in murine diabetic wounds (Khanna et al., 2010; Gallagher et al., 2015). Moreover, diabetic fibrocytes show a pro-inflammatory phenotype, which also contributes to arrest the process at the inflammatory phase (Falanga, 2005).

In the migration-proliferation phase, oxidative stress exerts its effects on endothelial cells, keratinocytes and fibroblasts, resulting in endothelial dysfunction, abnormal keratinocyte migration and proliferation, as well as impaired fibroblast proliferation, migration, differentiation. Oxidative stress is commonly implicated as an important unifying mechanism in endothelial dysfunction, which underlies both the micro- and macrovascular complications of T2DM (Rochette et al., 2014). Oxidative stress is a cell damage related factor that causes damage to proteins, lipids, and DNA in the cells, leading to cell death and subsequent tissue dysfunction (Long et al., 2016). Oxidative stress alters the functional capacity of endothelial nitric oxide synthase (eNOS) and directly degrades vasoprotective nitric oxide (NO) by ROS, resulting in diminished bioavailability of NO and endothelial dysfunction (Roberts and Porter, 2013). Oxidative stress also impairs keratinocytes. High-glucose environments disturb keratinocyte function including migration and proliferation. Gap junction abnormalities increase oxidative stress (Hu and Lan, 2016). Higher oxidative stress is associated with increased production of Interleukin (IL)-8 in high glucose–treated keratinocytes, which is responsible for recruiting neutrophils and impaired wound healing (Lan et al., 2013). Oxidative stress contributes to antiangiogenic molecule thrombospondin-1 (TSP1) DNA hypomethylation in keratinocytes exposed to hyperglycemia, resulting in overexpression of TSP1 that impairs proper wound healing (Lan et al., 2016). Oxidative stress also impairs fibroblasts. Increased free radical generation and lack of antioxidant defenses interfere with fibroblast proliferation (Schafer and Werner, 2008). Diabetic fibrocytes also demonstrate reduced expression of the C-X-C motif and C-C motif chemokine receptors (CXCR)4, (CCR)5, and CCR7 with weak migration in response to their ligands (CXCL)12, (CCL) 5, and CCL21 (Walker et al., 2018). Diabetic fibrocytes lose the ability to differentiate into myofibroblast-like cells on stimulation by growth factors that promote wound healing (Walker et al., 2018).

In the remodeling phase, oxidative stress impairs collagen deposition and ECM remodeling. Delayed diabetic wound healing is characterized by an increase in matrix metalloproteinase (MMPs) and a reduction in some growth factors, such as TGF-β1 (Jude et al., 2002; Blakytny and Jude, 2006; Long et al., 2016). TGF-β1 signaling works as the regulator in a series of biological processes of skin regeneration and wound healing, such as angiogenesis and re-epithelialization (Amento and Beck, 1991). High levels of ROS impair collagen production and tissue growth, resulting in halted wound closure (Loo et al., 2012). Interference of ECM production is highlighted through disruption of TGF-β1 signaling (Davies, 2005; He et al., 2014). High ROS levels affect ECM remodeling by regulating MMP expression (Hantke et al., 2002), which remodels tissue through proteolytic degradation (Caley et al., 2015). Excessive MMP activity inhibits the remodeling process and delays wound healing (Ravanti and Kahari, 2000).

Taken together, the impact of oxidative stress induced by hyperglycemia is widespread impairing the majority of cells and mechanisms involved in wound healing.

## Antioxidant Therapy in Diabetic Wound Healing

### The Nrf2 Pathway and Antioxidant Therapy

The nuclear factor erythroid–related factor 2 (Nrf2)/kelch-like erythroid cell–derived protein 1 (Keap1) pathway encompasses a central cellular defense mechanism that maintains redox homeostasis, but is disrupted in T2DM (Kensler et al., 2007; Soares et al., 2016). Nrf2 is a transcription factor regulating a wide range of genes influencing redox homeostasis, metabolism and repair (Hayes and Dinkova-Kostova, 2014). Keap1 is a Cul-E3 ligase regulating the progress of Nrf2 turnover and the ability of Nrf2 to translocate to the nucleus and activate the cellular antioxidant response (Wakabayashi et al., 2004). Keap1 functions as an intracellular ROS sensor and oxidants and electrophiles modify its cysteine residues. Under unstressed conditions, Keap1 interacts with Nrf2 and the cell’s actin cytoskeleton to sequester Nrf2 to the cytoplasm and promote ubiquitination and degradation of Nrf2 (Choi et al., 2014; Zoja et al., 2014). In the presence of oxidative stress, certain cysteine-rich oxidant and electrophile sensing regions of Keap1 are covalently modified, preventing Nrf2 ubiquitination. Nrf2 dissociates from its repressor Keap1, translocating to the nucleus and forming heterodimers with the Maf protein in the nucleus binding to Maf recognition element sequences, such as the antioxidant response element (ARE) and electrophile response element (EpRE) (Surh et al., 2005). Nrf2 responds to oxidative stress by activating key genes including NAD(P)H quinone oxidoreductase 1 (NQO1), manganese superoxide dismutase (MnSOD), heme oxygenase 1 (HO-1), glutamate cysteine ligase (GCL), and glutathione S-transferases (GSTs) (Baird and Dinkova-Kostova, 2011; Ruiz et al., 2013).

Since the Nrf2/Keap1 pathway is one of the master switch systems in oxidative stress (Sies, 2015), therapies based on Nrf2/Keap1 pathway have been reported. Keap1 is an important target in antioxidant therapy. Topical delivery of siRNAs is considered to be effective in diabetic wound treatment due to its temporary limitation to local applications and extrachromosomal function. Soares et al. (2016) reported that application of siRNA against Keap1 improved the redox homeostasis and accelerated diabetic tissue regeneration to near-normal levels by upregulating Nrf2-downstream antioxidant gene products such as NQO1 and MnSOD. Biomaterials designed to improve delivery efficiency of siKeap1 also show excellent effect in diabetic wound healing with severe oxidative stress. Rabbani et al. (2017) constructed a novel lipoproteoplex (LPP) nanoparticle, with optimal siRNA complexation, minimal cytotoxicity, and increased transfection efficacy. Application of this LPP complexing siKeap1 restored Nrf2 antioxidant function, accelerated diabetic tissue regeneration, and augmented reduction-oxidation homeostasis in murine diabetic wounds. Together, these studies suggest that targeted therapeutic strategies that reduce Keap1 expression may be an ideal, rapidly translatable way to treat cutaneous defects in T2DM.

Nrf2 is a central regulator of redox mechanisms. Firstly, topical application of Nrf2 activators provides a practical therapeutic intervention in diabetic wound healing. Sulforaphane (SF) and cinnamaldehyde (CA) are two known activators of NRF2 (Wu et al., 2013, 2016; Jiang et al., 2014), shown to be effective in ameliorating diabetic wound healing in a mouse model (Long et al., 2016). Likewise, Li et al. (2018b) showed that Dimethyl fumarate (DMF), a potent activator of NRF2, minimized oxidative damage and inflammation, and accelerated diabetic wound healing by producing DMF-elevated antioxidants and neutralizing ROS-induced excessive free radicals. Improving Nrf2 expression level in diabetic wound tissue is also a promising therapeutic strategy. RTA 408, a semi-synthetic oleanane triterpenoid, has been shown to have cytoprotective effects in human and rat skin through upregulation of Nrf2 target genes (Reisman et al., 2014, 2015). Rabbani et al. (2018) reported that application of 0.1% RTA 408 led to accumulation of Nrf2 in the nucleus, in conjunction with induction of the key antioxidant NQO1, and reversed the delayed healing trend of full thickness cutaneous wounds, significantly reducing the time to re-epithelialization in Leprdb/db mice. Genistein, an isoflavone in legumes with estrogen-like and antioxidative effects, accelerated wound healing in diabetic ICR mice by elevating Nrf2 and its related markers such as HO-1, GPx, catalase, and SODs (Wang et al., 1996; Eo et al., 2016). Additionally, carriers with Nrf2 have been shown to be excellent therapeutic strategies in diabetic wound healing. Li et al. (2018a) isolated the exosomes from Nrf2 overexpression adipose-derived stem cells (ADSCs). Compared with exosomes that originated from wild-type ADSCs, treatment with exosomes derived from ADSCs overexpressing Nrf2 were more effective in increasing granulation tissue formation, angiogenesis, and reducing inflammation and oxidative stress-related proteins detected in the wound beds of diabetic rats (Li et al., 2018a). Therefore, Nrf2 is a pertinent therapeutic target in diabetic wound healing.

**FIGURE 1:**
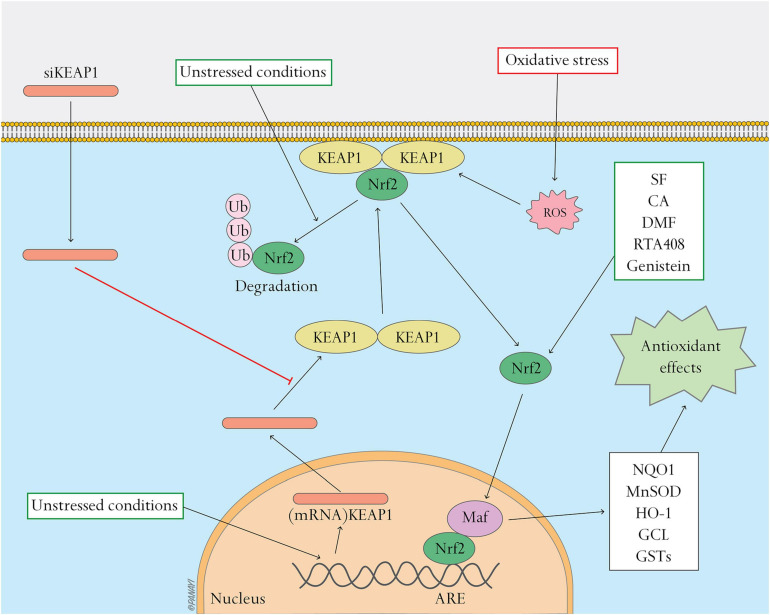
Nrf2 pathway and related antioxidant therapy. Nrf2 pathway and related antioxidant therapy. This figure shows the activation of the Nrf2 pathway and effects of antioxidant therapy targeting this pathway. Under unstressed conditions, Keap1 interacts with Nrf2 and the cell’s actin cytoskeleton to keep Nrf2 inactive in the cytoplasm and promote ubiquitination and degradation of Nrf2. Oxidative stress causes Nrf2 to detach from Keap1 and translocate to the nucleus where it heterodimerizes with Maf. The Nrf2-Maf heterodimer binds to ARE to induce the expression of antioxidant and metabolic genes including NQO1, MnSOD, HO-1, GCL, and GSTs. Oxidative stress can be regulated by activating the Nrf2 pathway. siKeap1 downregulates the levels of Keap1 by incorporating into an RNA-induced silencing complex (RISC) and inducing degradation of the complementary mRNA of Keap1. Nrf2 activators, such as SF, CA, DMF, RTA408 and genistein, stimulate the Nrf2 pathway and ameliorate oxidative stress.

Studies reveal the importance of the Nrf2/Keap1 pathway in antioxidant use in diabetic wound healing. The aforementioned studies provide insight that Nrf2 and Keap1 can be significant targets in gene and molecular therapy of diabetic wound healing (Figure 1).

### The NFκB Pathway and Antioxidant Therapy

The nuclear factor κB (NFκB) signaling pathway is known for its pro–inflammatory and pro–oxidant functions, and is reported as a master switch system in oxidative stress (Sies, 2015). Oxidative stress as well as inflammation activate the NFκB complex, which in turn is related to the cellular redox state (Hirota et al., 1999). Increasing expressions of NFκB genes and proteins contribute to enhanced oxidative stress (Mariappan et al., 2010).

The NFκB is a transcription factor consisting of homo- and heterodimers of five distinct proteins, the REL subfamily proteins (p65/RELA, RELB, and c-REL) and the NFκB subfamily proteins (p50 and p52) (Mitchell et al., 2016; Lingappan, 2018). NFκB is normally localized in the cytoplasm as a heterodimer such as p50/p65 (RelA). The Rel homology domain (RHD) in NFκB reacts on dimerization, recognition and binding to DNA as well as interaction with the inhibitory κB (IκB) proteins. IκB proteins bind to and block the nuclear localization signal of NFκB, resulting in sequestering of p65 in the cytoplasm and inhibiting the transcriptional activity of NFκB (Vallabhapurapu and Karin, 2009). IκB proteins possess nuclear export signals removing NFκB proteins from the nucleus, strictly regulating the pathway. Inflammatory signals (such as TNF-α or lipopolysaccharide) induce phosphorylation of IκB proteins by upstream kinases (IKK), resulting in the ubiquitination and degradation of IκB. Active NFκB translocates to the nucleus and activates the target genes (Zhang et al., 2017). NFκB induces the expression of inflammatory cytokines such as tumor necrosis factor alpha (TNF-α) (Yao et al., 1997), interleukin 1 beta (IL-1β) (Goto et al., 1999), interleukin 6 (IL-6) (Libermann and Baltimore, 1990), cyclooxygenase 2 (COX-2) (Kim et al., 2000), and inducible nitric oxide synthase (iNOS) (Taylor et al., 1998). The NFκB pathway also induces NADPH oxidase (NOX-2) subunit gp91phox and exacerbates oxidative stress (Anrather et al., 2006). Studies have reported that expression of NFκB pathway genes is upregulated in diabetic rats, triggering downstream expression of inflammatory cytokines (such as TNF-α, IL-1β, and IL-8) and delaying wound healing (Ren et al., 2019). NFκB also regulates the NOD-like receptor protein-3 (NLRP3) inflammasome (Bauernfeind et al., 2009), which is a target mechanism in promoting wound healing during the early stages (Weinheimer-Haus et al., 2015). It should be noted that although the NFκB pathway also has antioxidant functions and targets such as MnSOD (Kairisalo et al., 2007; Lingappan, 2018), current therapeutic strategies focus on suppression of the NFkB pathway in diabetic wound healing. NFκB has been considered as a crucial molecule contributing to end-organ damage T2DM. Therapeutic strategies related to the NFκB pathway are as follows.

Application of miR-146a against the NFκB pathway in wound healing has been reported. miR-146a is an anti-inflammatory microRNA known for its inhibitory effect on the NFκB pro-inflammatory signaling pathway, known to be decreased in diabetic wound healing (Xu et al., 2012). miR-146a targets and represses tumor necrosis factor receptor-associated factor 6 (TRAF6) and interleukin-1 receptor-associated kinase 1 (IRAK1), key adapter molecules in NFκB pathway activation (Xu et al., 2012; Xie et al., 2018). Enhancing expression of miR-146a can decrease levels of phosphorylated IκB-α, phosphorylated NFκB and total NFκB (Chen et al., 2013). miR-146a in conjugation with cerium oxide nanoparticles (CNP) has been proposed as a therapy for diabetic wound healing. CNP-miR146a improved wound healing in a murine and porcine diabetic wound model (Zgheib et al., 2019). CNP-miR146a combined with an efficient delivery system, such as nanosilk (Niemiec et al., 2020) or self-healable zwitterionic cryogels (Sener et al., 2020), demonstrated efficacy in accelerating diabetic wound healing.

SIRT1 (Sirtuin 1) is an important target in diabetic wound healing, and antagonizes the effect of NFκB pathway. SIRT1, a NAD-dependent class III histone deacetylase, suppresses binding of NFκB to inflammation-related gene promoters and its transcriptional activities by deacetylating the p65 subunit at lysine (Yeung et al., 2004). Downregulation of SIRT1 leads to hyperacetylation of p65, resulting in inflammation (Yeung et al., 2004; Kauppinen et al., 2013). Low expression of SIRT1 has been shown in diabetic rats and human umbilical vein endothelial cells (HUVECs) under hyperglycemia conditions (Yerra et al., 2017; Li et al., 2019). Therapeutic strategies of diabetic wound healing targeting the SIRT1 have been reported in recent years. Resveratrol, a well-known SIRT1 activator, accelerates wound healing by attenuating oxidative stress-induced impairment of cell proliferation and migration (Zhou et al., 2021). SIRT1 inhibits the expression of matrix metalloproteinase-1 and −3, delaying wound healing. Likewise, SIRT1 is a target of berberine, an eminent traditional Chinese and Ayurvedic medicine (Feng et al., 2019). Zhang et al. (2020) reported a novel berberine nanohydrogel that activates SIRT1, inhibiting the expression of NFκB and ameliorating diabetic wound healing through effective reduction of inflammation and promotion of angiogenesis. SRT1720, a specific SIRT1 activator, demonstrating locally improved wound healing and angiogenesis in STZ-induced diabetic mice (Li et al., 2019).

Several plant medicines that promote diabetic wound healing by regulating the NFκB pathway have been reported in recent years. Asiatic acid, the most active substance in the extracts of Chinese herbal compound Centella asiatica (Coldren et al., 2003), attenuates prolonged inflammation by downregulating NFκB activation in RAW 264.7 macrophage cells and reducing pro-inflammatory cytokine IL-1β, TNF-a and IL-6 (Yun et al., 2008). Han et al. (2019) reported that application of Asiatic acid together with aligned poly (L-lactic acid) electrospun scaffolds as drug-delivery system can accelerate re-epithelization, angiogenesis and ECM formation by relieving the high oxidative stress and inflammation in diabetic wounds. Likewise, syringic acid is involved in the suppression of NFκB activation and the inflammatory response to improve wound healing (Ren et al., 2019). Similarly, genistein promotes angiogenesis and accelerates healing related to the regulation of the NFκB pathway (Eo et al., 2016).

**FIGURE 2:**
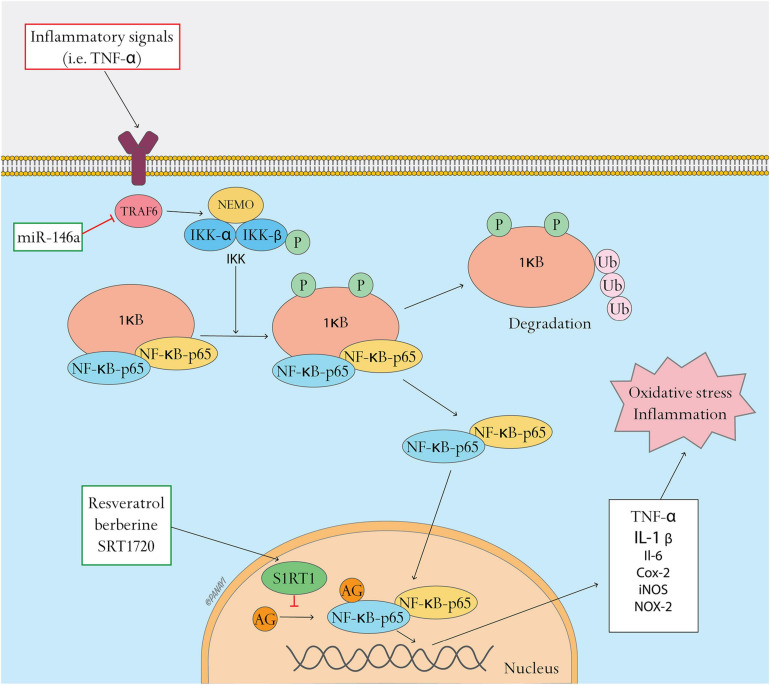
NFκB pathway and related antioxidant therapy. NFκB pathway and related antioxidant therapy. This figure shows the activation of the NFκB pathway and the effects of antioxidant therapy targeting this pathway. In resting state, NFκB dimers form a complex with the inhibitory protein IκB, which is located in the cytoplasm. Inflammatory signals (such as TNF-α) induce phosphorylation of IκB through upstream kinases (IKK), resulting in the ubiquitination and degradation of IκB. Active NFκB translocates to the nucleus and activates target genes including TNF-α, IL-1β, IL-6, COX-2, iNOS, and NOX-2, resulting in oxidative stress and inflammation. Oxidative stress can be regulated by inhibiting the NFκB pathway. MiR-146a can target and repress tumor necrosis factor receptor-associated factor 6 (TRAF6), inhibiting the activation of IKK and the NFκB pathway. SIRT1 activators, such as resveratrol, berberine and SRT1720, suppress binding of NFκB to inflammation-related gene promoters and their transcriptional activities by activating SIRT1, a NAD-dependent class III histone deacetylase that leads to deacetylation of the p65 subunit.

In summary, overactivation of NFκB pathway impairs wound healing in T2DM. Recent studies demonstrate that miR-146a and SIRT1 can be therapy targets in suppression of activated NFκB pathway and promotion of diabetic wound healing (Figure 2).

### Vitamins and Other Endogenous Molecules

Endogenous molecules such as glutathione, ubiquinones, uric acid, and lipoic acid, as well as vitamins E and C (ascorbic acid), carotinoids, and phenolic compounds have direct antioxidant effects and are involved in the regulation of the redox balance in skin wound healing (Schafer and Werner, 2008). As oxidative stress plays a major role in impaired diabetic wound healing, use of antioxidants to improve healing by modulating inflammation and the antioxidant system with no effect on glycemia is promising. Use of antioxidant vitamins, such as Vitamins E and C, has been reported to promote diabetic wound healing in recent studies. Vitamin E (α-tocopherol) interacts directly with peroxyl radicals, superoxide and singlet oxygen to protect cell membranes against lipid peroxidation, while vitamin C recycles the tocopherol radical, enhances its antioxidant activity, and generates dehydroascorbic acid to reduce oxidative stress (Maritim et al., 2003). Oral administration of vitamins E and C effectively restored the antioxidant enzyme activities, reducing ROS levels and accelerating wound closure in diabetic mice (Pessoa et al., 2016). Likewise, tocotrienols belong to the vitamin E-family. A study has reported that mono-epoxy-tocotrienol-α showed beneficial effects on wound healing in a diabetic (db/db) mouse model by increasing the expression of genes involved in cell growth, motility, angiogenesis, and mitochondrial function (Xu et al., 2017). Similarly, folic acid, a vitamin with direct antioxidant effects, improved collagen deposition and promoted wound healing via suppression of oxidative stress in diabetic mice (Zhao et al., 2018). Together, these studies indicate the importance of antioxidant vitamins in diabetic wound healing.

Overproduction of mitochondrial ROS (mtROS) is linked to the damaging effects of hyperglycemia (Giacco and Brownlee, 2010), and mitochondria-targeted antioxidants can be helpful in diabetic wound healing. Demyanenko et al. (2017) reported that administration of the mitochondria-targeted antioxidant SkQ1 suppressed oxidative stress, accelerated wound closure and stimulated epithelization, granulation tissue formation, and angiogenesis.

Therefore, administration of vitamins and other endogenous antioxidant molecules is an effective therapy method in diabetic wound healing that should be considered.

### Antioxidant Enzymes

Antioxidant enzymes play a crucial role in oxidative stress and chronic diabetic wounds (Panayi et al., 2020). Major antioxidant enzymes include superoxide dismutase (SOD), catalase, glutathione peroxidase, and heme oxygenase. In recent years, studies have reported the application of antioxidant enzymes in diabetic wound healing.

Superoxide dismutase is an antioxidant enzyme involved in oxidative stress. As an endogenous factor capable of scavenging free radicals, SOD catalyzes the decomposition of superoxide radicals into hydrogen peroxide which is converted into water and oxygen (Maier and Chan, 2002). However, SOD is expressed at inadequate levels in diabetic wound healing, resulting in excessive oxidative stress and impaired wound healing. In recent years, studies have reported use of SOD in diabetic wound healing, with SOD-loaded effectively promoting the repair of chronic diabetic wounds (Zhang et al., 2018).

HO-1, which is involved in the production of antioxidant enzymes associated with Nrf2 activation (Lee et al., 2011), is an important therapeutic target. HO-1 has protective effects on various cells and tissues while induction of HO-1 is a crucial event in the defense against cellular stress by maintaining anti-oxidant/oxidant homeostasis (Choi and Alam, 1996; Molavi and Mehta, 2004; Abraham and Kappas, 2008). HO-1 cleaves the α-methene bridges of heme to produce equimolar amounts of biliverdin and carbon monoxide (CO) (Maines and Kappas, 1977). Under the catalyzation of NADPH biliverdin reductase, biliverdin is transformed into bilirubin. Both bilirubin and biliverdin are powerful antioxidants (Jansen et al., 2010). CO functions as a gaseous signaling molecule that elicits an anti-inflammatory and anti-oxidative response (Ryter and Choi, 2009). Studies have shown the effect of HO-1 regulation in diabetic wound healing. Chen et al. (2016b) reported that use of hemin, a strong inducer of HO-1, can accelerate wound closure by reducing inflammatory cytokines such as TNF-α and IL-6, increasing antioxidants, and promoting angiogenesis in diabetic rat wounds. On the other hand, application of in-protoporphyrin IX (SnPPIX), a HO-1 inhibitor, exacerbated the oxidative stress conditions and delayed wound contraction in non-diabetic mice (Kumar et al., 2019). Together, studies in recent years have confirmed the efficacy of HO-1 regulation in diabetic wound healing.

Overall, regulation of the expression and activity of antioxidant enzymes is a promising therapy for diabetic wound healing.

### Hormones

Some hormones have been reported to be useful in diabetic wound healing. 17β-estradiol (E2) regulates energy homeostasis and glucose metabolism, which play a crucial role in regulating antioxidant enzyme expression and redox states (Bellanti et al., 2013). E2 can act as a cytoprotective antioxidant and has been shown to reduce excessive ROS formation, facilitating cell survival in a high glucose environment. Transplantation of hUCB-MSCs combined with E2 treatment can promote wound healing and angiogenesis through ERα-induced Nrf2 and Sirt3 signaling (Oh et al., 2019). Similarly, 5α-dihydrotestosterone has a positive antioxidant effect in secondary wound healing in diabetic rats, increasing the proportion of type I and type III collagen fibers and the level of SOD (Goncalves et al., 2016).

### Medicinal Plants

Medicinal plants, as well as their preparations and active compounds, have been utilized in wound healing and have been shown to regulate different metabolic pathways (Fronza et al., 2009; Kant et al., 2015). Some medicinal plants and their active compounds can modify the processes in the redox signaling pathway promoting diabetic wound healing. Medicinal plant compounds such as genistein and dimethyl fumarate accelerate wound healing through stimulation of the Nrf2 pathway and through other anti-inflammatory and antioxidant properties (Eo et al., 2016; Long et al., 2016). Medicinal plant compounds such as asiatic acid, syringic acid, and genistein accelerate the healing process through downregulation of the NFκB pathway (Eo et al., 2016; Han et al., 2019; Ren et al., 2019). Hydroethanolic extract of the strychnos pseudoquina has been shown to accelerate wound healing through modulating the oxidative status and microstructural reorganization in diabetic rats (Sarandy et al., 2017). Deoxyshikonin, the major angiogenic compound extracted from Lithospermi Radix, can exert antioxidant activities and promote phosphorylation of ERK and p38 and VEGFR-2 expression in diabetic wound healing (Park et al., 2018). Quercetin, a flavonoid plant ingredient, enhances organized granulation and fibrillar collagen formation by limiting prolonged inflammation, improving the glycemic state, increasing the insulin level, suppressing oxidative stress and enhancing the antioxidant defense system (Ahmed et al., 2018).

The application of medicinal plants in diabetic wound healing is a therapeutic strategy that has garnered attention in studies of recent years as using alternative therapies and natural remedies in faster wound healing upsurges in recent years. Medicinal plants and their active compounds with anti-inflammatory and antioxidant properties demonstrate a positive effect on diabetic wound healing.

### Biological Materials in Antioxidant Therapy

#### Hydrogels

Hydrogel is a promising material for wound healing, which possesses air permeability, moisture retention, possibility to load bioactive agents and the capability to absorb wound exudate and cool the wound surface leading to pain relief for patients (Gong et al., 2013; Zhang et al., 2018). Application of antioxidant-loaded hydrogels in diabetic wound healing has been developed. Superoxide dismutase (SOD)-loaded hydrogels have been shown to be effective in decreasing ROS generation and oxidative stress in chronic wounds. Zhang et al. (2018) constructed a novel SOD-loaded antioxidant hydrogel which exhibited sustained release of SOD with high activity in diabetic wound healing. The SOD-loaded hydrogel promoted healing of diabetic wounds by accelerating re-epithelialization and increasing collagen deposition. Similarly, Augustine et al. (2021) developed a biodegradable gelatin methacryloyl (GelMA) hydrogel patch incorporated with cerium oxide nanoparticles, which showed adequate exudate uptake capacity, effective free radical scavenging activity and capacity to establish a suitable microenvironment for cell proliferation in the wound healing of diabetic rats. Likewise, Alginate hydrogels have been particularly promising for use in wound healing owing to their good biocompatibility and ease of gelation (Lee and Mooney, 2012). Fan et al. (2019) developed a nanocomposite alginate hydrogel eluding the radical free scavenger edaravone with use of Eudragit nanoparticles to enhance the Edaravone’s solubility and stability. The Edaravone-eluting nanocomposite hydrogel downregulated ROS levels in diabetic mice and promoted wound healing in a dose-dependent manner. Sener et al. (2020) constructed a biomaterial system of zwitterionic cryogels laden with the anti-inflammatory and antioxidant CNP-miR146a, demonstrating efficacy and viability in the wound healing of diabetic mice. Taken together, antioxidant-loaded hydrogels are feasible therapies in diabetic wound healing.

#### Nanobiopolymers

Nanobiopolymers, such as nanocellulose, nanochitin, nanosilk, nanostarch, and microbial nanobiopolymers, which are produced by living organisms, have received widely scientific and engineering interests for their sustainable and biodegradable properties in recent years (Yang et al., 2019). Nanosilk, characterized by a high strength-to-density ratio and an ability to exhibit strain hardening, shows significant application in diabetic wound healing. Niemiec et al. (2020) prepared nanosilk-based materials on silk fibroin, a biocompatible polymer that can be fabricated into a nanostructure. The Nanosilk preparation showed effective delivery of CNP-miR146a to the wound bed, which downregulated pro-inflammatory signaling and promoted pro-fibrotic processes, ultimately accelerating diabetic wound healing.

#### Scaffolds

Scaffolds are regarded as promising materials for tissue engineering, and improved results have been reported in diabetic wound healing in studies of recent years. The 3D fibrous scaffolds of poly(lactic acid-co-glycolic acid) (PLGA) fabricated by liquid-collecting electrospinning have been used to simulate the ECM microenvironment, providing mechanical support for cell adhesion and promoting cell growth in wounds (Chen et al., 2016a). Han et al. (2019) utilized an asiatic acid -embedded aligned porous poly(L-lactic acid) electrospun fibrous scaffold (AA-PL), to promote angiogenesis, ECM formation and re-epithelization by relieving the high oxidative stress, inflammation and infection in diabetic wound healing.

#### Framework Nucleic Acids

Framework nucleic acids (FNAs) – biocompatible and biodegradable nucleic acids – show advantages in tailorable functionality and multiple modifiability, which can be applied to drug delivery and nanomedicine (Lin et al., 2020). Lin et al. (2020) demonstrated that framework nucleic acids (tFNAs) attenuated inflammation, prevented oxidative damage, and promoted angiogenesis in diabetic wound healing.

Therefore, studies in recent years have reported multiple combinations of antioxidant therapy and biological materials in diabetic wound healing. This provides insight that biological materials loaded with antioxidant agents can decrease oxidative stress and promote healing and are promising for diabetic wound healing.

## Conclusion and Prospects

Numerous studies have highlighted the promising potential of antioxidant therapy in diabetic wound healing given the central role of oxidative stress in the pathology of chronic diabetic wounds. This review summarizes the antioxidant therapy that has been studied for use in diabetic wound healing in the past 5 years. The NFκB and Nrf2/Keap1 pathways are key pathways in oxidative stress, therefore therapies targeting these pathways have been shown to effectively promote diabetic wound healing. Antioxidants and enzymes with direct antioxidant effects can downregulate oxidative stress and accelerate diabetic wound closure. Hormones can regulate energy homeostasis and glucose metabolism to relieve oxidative stress. Medicinal plants with anti-inflammatory and antioxidant properties have been shown to improve diabetic wound healing. Biological materials loaded with antioxidant agents decrease oxidative stress and promote diabetic wound healing. Despite the rapid development in the field of antioxidant therapy for diabetic wound healing, a complete cure for diabetic ulcers has yet to be developed. As the evidence from recent years is largely based on animal experiments, further research on the use of antioxidant therapy in humans, as well as the function of antioxidants at the genomic and molecular level, is necessary to elucidate the key roles of oxidative stress and redox homeostasis in diabetic wound healing.

## Author Contributions

FC, BM, and GL: conceptualization and funding acquisition. LC and YX: formal analysis. AA, YH, and CY: investigation. WZ, YS, ML, HX, LH, and CY: resources and supervision. XX and ZL: validation. WZ: visualization and writing (original draft). AP: writing (review and editing). All authors contributed to the article and approved the submitted version.

## Conflict of Interest

The authors declare that the research was conducted in the absence of any commercial or financial relationships that could be construed as a potential conflict of interest.
